# Die Unité Rhumatologique des Affections de la Main (URAM)-Skala und deren Gütekriterien bei der Dupuytren’schen Kontraktur: The Unité Rhumatologique des Affections de la Main (URAM) Scale and its Measurement Properties for Dupuytren’s Disease

**DOI:** 10.1055/a-2833-9848

**Published:** 2026-05-05

**Authors:** Sonja Elisabeth Pelzmann

**Affiliations:** 1 Doctoral Programme in Medical Science, Paracelsus Medizinische Privatuniversität, Salzburg, Austria; 2 Institut für Physikalische Medizin und Rehabilitation, Wiener Gesundheitsverbund Klinik Ottakring, Vienna, Austria

**Keywords:** M. Dupuytren, URAM-Skala, Validität, Reliabilität, Responsivität, Dupuytrenʼs disease, URAM scale, validity, reliability, responsiveness

## Abstract

**Hintergrund:**

Zur Messung von Einschränkungen werden bei der Dupuytren’schen Kontraktur (DK) unterschiedliche Fragebögen verwendet, unter anderem die Unité Rhumatologique des Affections de la Main (URAM)-Skala. Dieser erste Dupuytren-spezifische Fragebogen ist im deutschsprachigen Raum kaum bekannt oder gar in Verwendung. Ziel dieser Übersichtsarbeit ist es, die Gütekriterien der URAM-Skala aufzuzeigen und damit ihren Einsatz in der Klinik und Forschung im deutschsprachigen Raum zu etablieren.

**Methodik:**

Dieses Review wurde unter Berücksichtigung des COSMIN (COnsensus-based Standards for the selection of health Measuresment INstruments) methodology for systematic reviews of Patient-Reported Outcome Measures (PROMs) erstellt. In unterschiedlichen elektronischen Datenbanken (PubMed, CINAHL, Embase), spezifischen Journalen sowie zusätzlichen Quellen wurde nach relevanten und vorhanden Daten gesucht. Die inkludierten Publikationen wurden mittels COSMIN-Checklist und der Grading of Recommendations, Assessment, Development, and Evaluations (GRADE)-Methode analysiert.

**Ergebnisse:**

Die Ergebnisse der 9 eingeschlossenen Publikationen zeigten, dass die URAM-Skala über eine gute Konstruktvalidität (r=0,21–0,807), eine exzellente Retest-Reliabilität (ICC=0,35–0,96), eine exzellente interne Konsistenz (Cronbach’s Alpha=0,81–0,91), eine mittlere bis hohe Responsivität (ES: 0,56–0,96) sowie eine hohe Spezifizität (86–88%), aber niedrige Sensitivität (52–56%) bei der DK verfügt. Die Qualität der Evidenzen waren hinsichtlich Responsivität hoch, jene der Konstruktvalidität und Reliabilität moderat und die der Inhaltsvalidität von geringer Qualität.

**Schlussfolgerung:**

Diese Daten, die auf wenigen Publikationen basieren, zeigten, dass die gut akzeptierte und leicht anwendbare URAM-Skala als Entscheidungshilfe und Forschungstool bei dieser Erkrankung herangezogen werden kann.

## Abstract (en)

**Introduction:**

Various questionnaires are used to measure limitations in Dupuytren’s disease (DD), including the Unité Rhumatologique des Affections de la Main (URAM) scale. This first Dupuytren-specific questionnaire is hardly known or used in German-speaking countries. The aim of this review is to highlight the measurement properties of the URAM scale and thus establish its use in clinical practice and research in German-speaking countries.

**Methods:**

This review was conducted using the COSMIN methodology (COnsensus-based Standards for the selection of health Measurement INstruments) for systematic reviews of Patient-Reported Outcome Measures (PROMs). Relevant and available data were searched for in various electronic databases (PubMed, CINAHL, Embase), specific journals, and additional sources. The included publications were analyzed using the COSMIN checklist and the GRADE (Grading of Recommendations, Assessment, Development, and Evaluations) approach.

**Results:**

The results of the 9 included publications showed that the URAM scale has good construct validity (r=0,21–0,807), excellent retest reliability (ICC=0,35–0,96), excellent internal consistency (Cronbach’s alpha=0,81–0,91), moderate to high responsiveness (ES: 0,56–0,96), and high specificity (86–88%), but low sensitivity (52–56%) for DD. The evidence was high in terms of responsiveness, moderate in terms of construct validity and reliability, and of low quality in terms of content validity.

**Conclusion:**

However, these data, which are based on a small number of publications, show that the well-accepted and easy-to-use URAM scale can be used as a decision-making aid and research tool for this disease.

## Einleitung

Die Dupuytren’sche Kontraktur (DK) oder Morbus Dupuytren ist eine proliferative Erkrankung der Hand, die zu Einschränkungen der Handfunktion bei Tätigkeiten des täglichen Lebens, der Produktivität und der Freizeit führt. Darüber hinaus wird von den Betroffenen ein weitreichender Einfluss der Erkrankung auf sich und deren Leben wahrgenommen 1 2 3 4 . In der klinischen Praxis und Forschung werden zusätzlich zur Goniometermessung, die den Goldstandard der Erhebung der charakteristischen Extensionsdefizite darstellt 5 , unterschiedliche Fragebögen 6 7 verwendet. Fragebögen stellen eine wichtige Ergänzung zu traditionellen ärztlichen Evaluierungen dar. Sie sind Standard, wenn es darum geht, die Patientenperspektive, die subjektive Wahrnehmung der individuellen Bedürfnisse oder die Auswirkungen auf den Gesundheitszustand des Einzelnen zu erfassen 8 9 10 . Valide, reliable und responsive Fragebögen werden dazu verwendet, relevante Daten zu erheben, Diagnosen oder Prognosen zu erstellen, den Behandlungs- oder Therapiebedarf zu begründen oder den Effekt von Interventionen zu evaluieren 11 12 . Der erste spezifische Fragebogen für die Beurteilung der eingeschränkten Handfunktion aufgrund der DK, die „Rhumatologiques des Affections de la Main (URAM)“ -Skala, wurde im Lariboisière Hospital, Paris, Frankreich entwickelt. Sie besteht aus 9 Items, die mit 0 (ohne Schwierigkeit), 1 (mit geringer Schwierigkeit), 2 (mit etwas Schwierigkeit), 3 (mit großer Schwierigkeit), 4 (fast unmöglich) und 5 (unmöglich) bewertet und zu einem Gesamtwert addiert werden. Ein hoher Gesamtwert beschreibt folglich eine erhebliche subjektiv wahrgenommene funktionelle Beeinträchtigung oder Störung im Alltag und bei Aktivitäten des täglichen Lebens aufgrund der DK. Vorranging wird das Können bei der persönlichen Hygiene, beim Ergreifen von Gegenständen unterschiedlicher Größe oder Gesten erfragt 13 14 . Im Gegensatz zum Michigan Hand Outcomes Questionnaire (MHQ) 15 werden anhand der URAM-Skala die Hände gemeinsam evaluiert. Sie ist in englischer, französischer, deutscher ( Tab. 1 : Deutsche validierte Version der Unité Rheumatologique des Affections de la Main (URAM) Skala) 16 , niederländischer, italienischer, spanischer und koreanischer Sprache verfügbar 13 14 16 17 18 19 20 . Ziel dieser Studie war es, einen systematischen Überblick und eine Analyse der Qualität und Evidenzen der Gütekriterien der URAM-Skala bei der DK zu schaffen, um dessen Zweckmäßigkeit bei der Befundung, als Entscheidungshilfe bei Behandlungsoptionen bzw. -zeitpunkten sowie der Beurteilung von Behandlungseffekten bei dieser Erkrankung darzulegen und damit deren Einsatz im deutschsprachigen Raum zu etablieren.

**Table:** **Tab. 1** Deutsche validierte Version der Unité Rheumatologique des Affections de la Main (URAM)-Skala (Prof. Dr. Karsten Knobloch, Marie Kühn, Dr. Heiko Sorg, Prof. Dr. Peter M. Vogt, Med. Hochschule Hannover) 16.

Können Sie…	ohne Schwierigkeit (0)	mit geringer Schwierigkeit (1)	mit etwas Schwierigkeit (2)	mit großer Schwierigkeit (3)	fast unmöglich (4)	unmöglich (5)
1. sich mit einem Waschlappen selbst waschen und dabei die Hand flach halten?	□	□	□	□	□	□
2. sich das Gesicht waschen?	□	□	□	□	□	□
3. eine Flasche in Ihrer Hand halten?	□	□	□	□	□	□
4. jemanden per Handschlag begrüßen?	□	□	□	□	□	□
5. über etwas oder jemanden streicheln?	□	□	□	□	□	□
6. die Hände zusammenklatschen?	□	□	□	□	□	□
7. die Finger auseinanderspreizen?	□	□	□	□	□	□
8. sich auf Ihre Hand aufstützen?	□	□	□	□	□	□
9. kleine Gegenstände mit Daumen und Zeigefinger aufheben?	□	□	□	□	□	□

## Methode

Dieses Systematic Review wurde unter Berücksichtigung des COSMIN methodology for systematic Review of Patient-Reported Outcome Measures (PROMs) 21 durchgeführt [Bibr R2025-08-OA-1771-0022] . Die Daten wurden im Rahmen des PhD-Projektes von der Autorin des Artikels gesichtet, bewertet und aufbereitet. Die Ergebnisse wurden extrahiert und in Microsoft Word Datenblättern und Tabellen festgehalten.

**Abb. 1:**
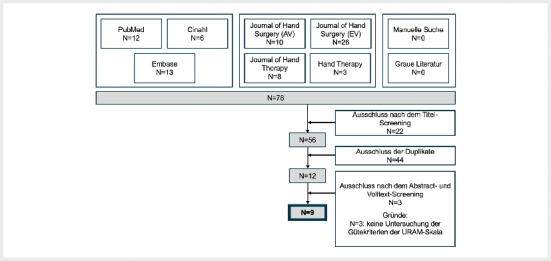
Suchstrategie; AV=American Volume; EV=European Volume.

In elektronischen Datenbanken (PubMed, Cinahl, Embase) und fachspezifischen Journalen (Journal of Hand Surgery (American Volume), Journal of Hand Surgery (European Volume), Journal of Hand Therapy, Hand Therapy,) wurde im Februar 2025 und einer zusätzlichen Suche im Februar 2026 systematisch unter der Verwendung von Kriterien, Schlüsselwörtern (,dupuytren*‘ [MeSH Terms], ,URAM‘, ,measurement property‘, ,validity‘, ,reliability‘ und ,responsiveness‘) und deren Kombination (,dupuytren*‘ [MeSH Terms] AND URAM (,measurement property‘ OR ,validity‘ OR ,reliability‘ OR ,responsiveness‘) nach vorhandenen und relevanten Informationen gesucht. Publikationen in englischer und deutscher Sprache, die zwischen Januar 2011 und Januar 2026 veröffentlicht wurden, die die URAM-Skala, deren Entwicklung oder Gütekriterien thematisierten und deren Patienten älter als 18 Jahre waren, wurden hierbei eingeschlossen. Studien, die nicht in englischer- oder deutscher Sprache verfasst waren, die Patienten mit anderen Erkrankungen (z. B. Karpaltunnelsyndrom, Sehnenverletzungen) inkludierten, wurden ausgeschlossen. Um mögliche zusätzliche Informationen zu identifizieren, wurden die Referenzlisten aller inkludierten Publikationen und graue Literatur herangezogen, sowie eine manuelle Suche durchgeführt. Reviews, Konferenz- sowie Workshop-Abstracts wurden in dieser Übersichtsarbeit nicht berücksichtigt ( Abb. 1 : Suchstrategie). Die methodische Qualität der eingeschlossenen Studie wurde zuerst mittels der COnsensus-based Standards for the selection of health Measurement INstruments (COSMIN) Risk of Bias Checkliste analysiert. Sie besteht aus 10 Boxen, wobei jede ein anderes Gütekriterium (Entwicklung (Patient Reported Outcome Measurement (PROM) development), Inhaltsvalidität (content validity), Strukturvalidität (structural validity), interne Konsistenz (internal consistency), kulturelle Validität (cross-cultural validity / measurement invariance), Reliabilität (reliability), Messfehler (measurement error), Kriteriumsvalidität (criterion validity), Hypothesen (Hypotheses testing for construct validity) und die Responsivität oder Veränderungssensitivität (Responsiveness) evaluiert 23 . Im Anschluss erfolgte die qualitative Einschätzung der Messeigenschaften unter Zuhilfenahme der aktualisierten Kriterien für gute Gütekriterien von Prinsen et al. 24 , die auf den Kriterien von Terwee et al. 25 und Prinsen et al. 26 basieren. Die Qualität der Evidenz wurde anhand der Grading of Recommendations, Assessment, Development, and Evaluations (GRADE) – Methode 27 evaluiert. Die folgenden Daten wurden von jeder eingeschlossenen Studie erfasst: Autor(en), Jahr der Veröffentlichung, Studienpopulation (Fallzahl, Geschlecht, Alter), erhaltene Interventionen, untersuchte Gütekriterien, Setting und Land sowie verwendete Sprache.

## Ergebnisse

Nach der Durchsicht der elektronischen Datenbanken, der relevanten Journale und der zusätzlichen Quellen konnten 78 Treffer identifiziert werden. Unter Berücksichtigung der Ein- und Ausschlusskriterien wurden 22 Publikationen im Rahmen des Titel-Screenings exkludiert, da sie keine Informationen über die URAM-Skala oder deren Gütekriterien bei der DK enthielten. In weiterer Folge wurden 44 Publikationen exkludiert, da es sich hierbei um Duplikate handelte. Nach dem Abstract- und Volltext-Screening wurden 3 weitere Publikationen ausgeschlossen, da diese die Gütekriterien der URAM-Skala nicht untersuchten. Letztendlich konnten 9 Publikationen in dieses Review eingeschlossen werden.

Die 9 inkludierten Studien 13 14 17 19 20 28 29 30 31 wurden zwischen 2011 und 2023 veröffentlicht und umfassten eine Stichprobengröße von 53 13 30 – 231 17 Patienten im Alter zwischen 34 29 und 90 29 Jahren. Der Anteil der Männer in den Studien reichte von 63% 29 bis 85% 14 . Die Betroffenen erhielten im Vorfeld keine Intervention 14 19 , wurden konservativ 20 , mittels verschiedener chirurgischer Techniken 17 20 28 29 oder mittels Kollagenase (Collagenase Clostridium Histolyticum (CCH)-Injektion) 17 31 behandelt. In 2 Studien 13 30 wurde nicht berichtet, welche Behandlungsoption angewendet wurde. Die Studien wurden in Kliniken 13 17 19 20 30 31 , Handzentren 28 29 oder Privatpraxen 14 verschiedener Länder und in unterschiedlichen Sprachen durchgeführt ( Tab. 2 : Charakteristik der eingeschlossenen Studien).

**Table:** **Tab. 2** Charakteristik der inkludierten Studien.

Referenz	Fallzahl	Geschlecht	Alter (mean) (SD, Weite)	Charakteristik der DK-Präsentation / Behandlung	Untersuchte Gütekriterien	Land	Sprache
Lee et al., 2024 20	N=59	Männer=83,3%	63,5 Jahre (39–81)	DK-Patient*innen, die entweder eine konservative- oder chirurgische Behandlung erhalten haben.	Kulturelle Validität der URAM-Skala	Korea	Koreanisch
					Reliabilität		
					Interne Konsistenz Retest-Reliabilität		
					Konstruktvalidität der URAM-Skala anhand von Korrelationen mit		
					Tubiana-Klassifizierung PRWE DASH oder Quick-DASH MHQ oder briefMHQ		
Sanjuan-Cervero et al., 2022 31	N=92	Männer=79,3%	68,07 Jahre (39–85)	DK-Patient*innen, die mittels CCH-Injektion behandelt wurden	Konstruktvalidität der URAM-Skala anhand von Korrelationen mit	Spanien	Englisch
					URAM SDSS briefMHQ PEM Kontraktur vor der Behandlung in MCPs und PIPs / Goniometermessungen Kontraktur nach der Behandlung in MCPs und PIPs / Goniometermessungen		
					Reliabilität		
					Retest-Reliabilität Interne Konsistenz		
					Responsivität		
					Minimal important Difference (MID)		
Lanfranchi et al., 2021 14	N=96	Männer=85%	66,8±9,3 Jahre	DK-Patient*innen, die keine handchirurgische Behandlung in den vorangegangenen sechs Monaten erhalten haben	Kulturelle Validität der URAM-I (10)-Skala	Italien	Italienisch
					Konstruktvalidität der URAM-Skala anhand von Korrelationen mit		
					SF36 HADS VAS		
					Konvergentvalidität der URAM-Skala anhand von Korrelationen mit		
					PRWHE		
					Strukturvalidität der URAM-I (10)-Skala (Faktorenanalyse)		
					Reliabilität		
					Retest-Reliabilität Interne Konsistenz		
Hensler et al., 2020 17	N=231 (273 Fälle)	Männer=79%	67 Jahre (SD 9)	DK-Patient*innen, die entweder eine CCH-Injektion oder eine chirurgische Behandlung (z. B. eine partielle Aponeurektomie und Knotenentfernung) erhalten haben	Konstruktvalidität in Korrelation mit MHQ	Schweiz	Deutsch
					EQ-5D-5l (deutsche Sprache) Flexionskontraktur in MCP, PIP, DIP / Goniometermessung		
					Strukturvalidität (Faktorenanalyse)		
					Reliabilität		
					Interne Konsistenz der URAM-Skala		
					Responsivität		
					Interpretation		
					Minimal important change (MIC) Minimal important difference (MID)		
Broekstra et al., 2018 19	N=193	Männer=65%	66,1 Jahre (SD 10,7)	DK-Patient*innen, die keine Behandlung erhalten haben	Gleichzeitige Validität	Niederlande	Niederländisch
					Reliabilität		
					Retest-Reliabiltät Interne Konsistenz Messfehler der URAM-Skala		
					Responsivität		
					Bodeneffekt /Floor effect		
					Interpretation		
Rodrigues et al. 2017 28	N=101	Männer=82%	67 Jahre (34–90)	DK-Patient*innen, die primär oder rezidivierend, die auf eine Fasziektomie oder Dermofasziektomie warten	Responsivität	Vereinigtes Königreich	Englisch
					Interpretation		
Rodrigues et al., 2015 29	N=110	Männer=63%	68 Jahre (34–90)	DK-Patient*innen, bei denen eine Fasziektomie oder Dermofasziektomie ansteht	Inhaltsvalidität der URAM-Skala	Vereinigtes Königreich	Englisch
Bernabé et al., 2014 30	N=53	Männer=83%	63 Jahre (SD 9)	DK-Patient*innen, nicht näher beschrieben	Konvergent (Konstrukt) Validität anhand von Korrelationen mit	Frankreich	Französisch
					Tubinana-Klassifizierung VAS (bei ADLs) CHFS DASH		
					Mean response time (MRT)		
Beaudreuil et al., 2011 13	N=53	Männer=83%	63,2±8,9 Jahre	DK-Patient*innen, nicht näher beschrieben	Inhaltsvalidität der URAM-Skala (Entwicklung)	Frankreich	Französisch
					Kulturelle Validität URAM (Englische Übersetzung)		
					Konstruktvalidität anhand von Korrelationen mit		
					Tubiana-Klassifizierung VAS – Einschränkung CHFS DASH VAS- Schmerz HADS Angst-Subskala HADS Depressions-Subskala		
					Reliabilität		
					Retest-Reliabilität Interne Konsistenz		
					Responsivität		
					Effektgröße / Effect size (ES)		
					Clinical important change (CIC)		

URAM: Unité Rhumatologique des Affections de la Main; SDSS: Southamptom Dupuytren’s Scoring Scheme; DASH: Disability of the Arm, Shoulder and Hand Questionnaire; QuickDASH: Quick Disability of the Arm, Shoulder and Hand Questionnaire, MHQ: Michigan Hand Outcomes Questionnaire; briefMHQ: brief Michigan Hand Outcomes Questionnaire; PEM: Patient Evaluation Measure; EQ-5D-5L: European Quality of Life 5 Dimensions 5 Level Version; PRWHE: Patient Rated Wrist/Hand Evaluation; PRWE: Patient-Rated Wrist Evaluation; SF-36: Short Form-36; CHFS: Cochin Hand Function Scale; HADS: Hospital Anxiety and Depression Scale; VAS: visuelle Analogskala; MCP: Metacarpophalangeal Gelenk; PIP: proximales Interphalangealgelenk; DIP: distales Interphalangealgelenk; SD: Standarddeviation; ES: Effect size; MIC: Minimal important change; MID: Minal important difference; CIC: Clinical important Change.

### 1. Methodische Qualität der Studien anhand der COSMIN Risk of Bias-Checklist und der Kriterien für gute Messeigenschaften

Die inkludierten Studien wiesen anhand der Risk of Bias-Checklist viermal einen globalen Wert von „sehr gut“/„very good“ (V) 17 28 30 31 , einmal „adäquat“/„adequate“ (A) 14 , zweimal „zweifelhaft“/„doubtfult“ (D) 19 29 und zweimal „inadäquat“/“inadequate“ (I) 13 20 auf. Die Qualität der Messeigenschaften der einzelnen Studien reichte von ausreichend bis unzureichend oder war unbestimmt. Jene der internen Konsistenz 13 14 17 19 20 31 war durchgehend ausreichend. Die Konstruktvalidität wurde mit ausreichend 14 17 oder unbestimmt 13 19 20 30 31 , die Reliabilität mit ausreichend 14 19 , unzureichend 31 oder unbestimmt 13 20 , sowie die Responsivität mit ausreichend 17 28 31 , unzureichend 19 oder unbestimmt 13 20 bewertet. Die Inhaltsvalidität wies eine unzureichende- 29 und unbestimmte 13 -, und der Messfehler 19 eine unzureichende Qualität auf. ( Tab. 3 : COSMIN Risk of Bias – Checkliste und Bewertung anhand der Kriterien für gute Messeigenschaften).

**Table:** **Tab. 3** COSMIN Risk of Bias – Checklist und Bewertung anhand der Kriterien für gute Messeigenschaften.

Referenzen	COSMIN Risk of Bias – Checklist: Bewertung pro Box*											Bewertung anhand der Kriterien für gute Messeigenschaften**								
	Box 1	Box 2	Box 3	Box 4	Box 5	Box 6	Box 7	Box 8	Box 9	Box 10	GLOBAL	Inhalts-validität	Strukturelle Validität	Interne Konsistenz	Reliabilität	Mess-fehler	Prüfung von Hypothesen zur Kontruktvalidität	Kulturelle Validität	Kriteriums-validität	Responsivität
Lee et al., 2023 20				V	I	D			A	A	**I**			+	?		?	?		?
Sanjuan-Cervero et al., 2022 31				V		V			V	V	**V**			+	–		?			+
Lanfranchi et al., 2021 14				V	A	V			V		**A**			+	+		+	?		
Hensler et al., 2020 17			V	V	V	V			V	V	**V**		+	+			+	?		+
Broekstra et al., 2018 19				V		D	D		V	V	**D**			+	+	–	?			–
Rodrigues et al., 2017 28										V	**V**									+
Rodrigues et al., 2015 29		D									**D**	-								
Bernabé et al., 2014 30									V		**V**						?			
Beaudreuil et al., 2011 13	D	D	A	V	I	A			I	I	**I**	?	?	+	?		?	?		?

Box 1: PROM Entwicklung, Box 2: Inhaltsvalidität, Box 3: strukturelle Validität, Box 4: interne Konsistenz, Box 5: kulturelle Validität, Box 6: Reliabilität, Box 7: Messfehler, Box 8: Kriteriumsvalidität, Box 9: Hypothesen, Box 10: Responsivität. *V=sehr gut / very good, A=adäquat / adequate, D=zweifelhaft / doubtful, I=Inadäquat / inadequate. **+=ausreichend / sufficient, -=unzuriechend / insufficient ?=unbestimmt / indetermindate.

### 2. Qualität der Evidenzen (GRADE)

Die Responsivität und Spezifizität wiesen eine hohe, die Konstruktvalidität, Reliabilität (Retest-Reliabilität und Interne Konsistenz) eine moderate, die Inhaltsvalidität und Sensitivität eine geringe Qualität der Evidenzen (GRADE) auf.

### 3. Gütekriterien

Am häufigsten wurden die Konstruktvalidität (N=7) 13 14 17 19 20 30 31 , gefolgt von der internen Konsistenz (N=6) 13 14 17 19 20 31 und der Responsivität (N=6) 13 17 19 20 28 31 untersucht. Lediglich die Kriteriumsvalidität wurde nicht untersucht.

### 3.1 Validität

Die Ergebnisse der Inhaltsvalidität belegten, dass 55% (154 von 278) der Probleme, die von Betroffenen genannten wurden, von der URAM-Skala nicht erfasst wurden 29 . Es wurde darauf hingewiesen, dass durch das Weglassen des Items #9 („Kleine Dinge mit Daumen und Zeigefinger aufnehmen“) der Anpassungsindex von 3,4 auf 2,8 verbessert wurde 17 . Die italienische Version (URAM-I(10)) wurde im Rahmen der Validierung um das Item #10 („Einen Handschuh an der betroffenen Hand anziehen“) ergänzt 14 Durch die Faktorenanalyse konnte in der italienischen Version der URAM-Skala eine 2-Faktoren-Struktur, „offene Hand“ und „geschlossene Hand“, identifiziert werden. Der Faktor für „offene Hand variierte hierbei zwischen 0,067 (Item #9) und 0,91 (Item #1), sowie für „geschlossene Hand“ zwischen 0,108 (Item #8) und 0,886 (Item #9) 14 . Die Konstruktvalidität, die in Summe gut war, wurde durch die Bewertung von Korrelationen zwischen der Tubiana-Klassifizierung oder anderen PROMs untersucht. Auf die höchsten Korrelationen und damit einem starken Zusammenhang wurde zwischen der URAM-Skala und der Southampton Dupuytren’s Scoring Scheme (SDSS), einem weiteren diagnose-spezifischen Fragebogen, (r=0,728–0,807) 31 , dem brief Michigan Hand Outcomes Questionnaire (briefMHQ) (r=0,682–0,76) 17 31 , der Tubiana-Klassifizierung (r=0,29–0,69) 17 20 30 31 , der visuellen Analogskala (VAS)-Einschränkung (r=0,67–0,69) 13 30 sowie dem Wert des Disability of the Arm, Shoulder and Hand Questiononnaire (DASH) (r=0,55) 13 hingewiesen. ( Tab. 4 : Konstruktvalidität der URAM-Skala in Korrelation mit den anderen PROMs). Bei der Evaluierung nach einem Jahr erreichten die Patienten einen Wert von 0, was auf einen erheblichen Bodeneffekt hinwies 17 .

**Table:** **Tab. 4** Konstruktvalidität der URAM-Skala in Korrelation mit den anderen PROMs.

Korrelation mit	SDSS	DASH	MHQ	briefMHQ	PEM	EQ-5D-5L	PRWHE	PRWE	PRWE – Schmerz	PRWE – Funktion	SF-36	CHFS	HADS – Angst	HADS – Depression	Tubiana / Kontraktur	VAS – Schmerz	VAS – Einschränkung
**URAM-Skala**	0,728–0,807	0,55	−0,76	0,682–0,76	0,649–675	0,46	0,56–0,69	0,56	0,46	0,61	0,21–0,3	0,634	0,26	0,05	0,29–0,69	0,26	0,67–0,69
**Referenz**	29	13	15	15 29	29	15	25	18	18	18	25	13	13	13	15 18 28 29	13	13 28

PROMs: Patient-reported outcome measures; URAM: Unité Rhumatologique des Affections de la Main; SDSS: Southamptom Dupuytren’s Scoring Scheme; DASH: Disability of the Arm, Shoulder and Hand Questionnaire; MHQ: Michigan Hand Outcomes Questionnaire; briefMHQ: brief Michigan Hand Outcomes Questionnaire; PEM: Patient Evaluation Measure; EQ-5D-5L: European Quality of Life 5 Dimensions 5 Level Version; PRWHE: Patient Rated Wrist/Hand Evaluation; PRWE: Patient-Rated Wrist Evaluation; SF-36: Short Form-36; CHFS: Cochin Hand Function Scale; HADS: Hospital Anxiety and Depression Scale; VAS: visuelle Analogskala.

### 3.2 Reliabilität

Die Retest-Reliabilität war bei kurzzeitigen Erhebungen (1–7 Tagen) exzellent (ICC=0,96–0,97) 13 14 , bei den mittelfristigen Erhebungen (bis zu 1 Monat) gering bis mäßig (ICC=0,35–0,519) 31 und bei den langfristigen Erhebungen (bis zu 1 Jahr) exzellent (ICC=0,89) 20 . Eine exzellente interne Konsistenz wurde beobachtet (Cronbach’s Alpha=0,81– 0,91) 13 17 20 31 .

### 3.3 Responsivität und Interpretierbarkeit

Die Effektgröße (Effect size (ES)) variierte (0,56–0,96) in den inkludierten Studien 13 17 20 28 . Im Vergleich der Baseline-Daten mit der 1-Monats-Folgemessungen zeigte sich hinsichtlich standardisiertem Antwortmittelwert (Standard Response Mean (SRM)) ein Wert von −1,136 31 . 2 Autoren 19 28 verwendeten die Receiver Operating Characteristic (ROC) Curve Analyse. Die Ergebnisse basierten bei Rodrigues et al. 28 auf der Global Rating of Change (GRC) mit einem Grenzwert (Cut-point) der Verbesserung von>10,5 28 . Die Fläche unter der Kurve (Area under the curve (AUC)) war 0,7 28 bzw. 0,67 19 . Die minimale wichtige Veränderung (Minimal Important Change (MIC)) reichte von 1,5 (SD±1,96) (Sensitivität: 52%, Spezifizität: 86%) 19 bis 10,5 (Sensitivität: 56%, Spezifizität: 88%) bei einer Fasziektomie oder Dermofasziektomie 28 . Bei einer Nadelfasziotomie war der MIC 2,9 (SD±2,6) 13 bzw. 6 17 nach einer Kollagenasebehandlung oder chirurgischen Behandlung. Der minimale wichtige Unterschied (Minimal Important Difference (MID)) war nach einer Kollagenasebehandlung oder chirurgischen Behandlung 7 17 und nach einer Fasziektomie oder Dermofasziektomie 8,3 (95% CIs: 0,04, 16,5) 28 .

## Diskussion

Ergebnisse dieses Reviews zeigen, dass die meisten Gütekriterien der URAM-Skala kaum bzw. die Kriteriumsvalidität nicht erforscht wurden. Lediglich eine Publikation evaluierte Aspekte der Validität und der Reliabilität der deutschen Fassung 17 . Die Ergebnisse belegen, dass die URAM-Skala über eine exzellente interne Konsistenz, eine gute Konstruktvalidität sowie mittlere bis hohe Responsivität verfügt. Unzureichende oder fehlende Informationen (z. B. hinsichtlich Hypothese 13 20 28 31 ) wirkten sich hierbei auf die Beurteilung Qualität der Messeigenschaften aus.

Die URAM-Skala misst die Funktionen bei Aktivitäten oder der Partizipation, die aufgrund der DK erschwert sind 8 . Bei der Item-Generation der Originalversion dienten die Aussagen von 85 Patienten und 7 Experten als Grundlage 13 . Die Übersichtarbeit von Pelzmann 4 , die die Aussagen über Probleme, Limitationen und Einschränkungen aufgrund der DK von 296 präsentiert, zeigt jedoch, dass diese Erkrankung einen weitreichenden Einfluss die Betroffen hat, und folglich als naheliegende Erklärung für die geringe Inhaltsvalidität herangezogen werden kann. Das „Handschuhe Anziehen“, das für Betroffene oftmals problematisch ist 4 32 , wurde weder in der Originalversion 13 , noch in der deutschen Version 16 aufgegriffen. Da dieses Fehlen durch die Ergebnisse von Rodrigues et al. 29 belegt und von verschiedenen Autoren 14 17 19 kritisiert wurde, wurde die italienische Version (URAM-I(10) 14 im Rahmen der Validierung um das Item #10 („Handschuh an der betroffenen Hand anziehen“/„Put a glove on the affected hand“) erweitert. Im Gegensatz dazu wird im Item #2 der SDSS das „Handschuhe anziehen“ erfragt. Darüber hinaus evaluiert die SDSS anhand ihrer 5 Items den möglichen Einfluss der DK auf die Betroffenen und deren Leben in den Bereichen „Unbehagen“, „persönliche Aktivitäten“ / „ADLs“, „häusliche Aktivitäten“ / „Haushalt“, „Arbeit“ / „soziale Aktivitäten“ und „Hobbies“ 4 33 . Eine deutsche validierte Version der SDSS ist zum jetzigen Zeitpunkt nicht existent. Anhand des Anpassungsindex zeigte sich, dass das Item #9 („Kleine Dinge mit Daumen und Zeigefinger aufnehmen“) unpassend und klinisch irrelevant ist 17 , da das Auftreten der DK in Fingerpaaren bei Daumen und Zeigefinger, mit 5,4% bei Männern und 3,7% bei Frauen, am niedrigsten ist 34 . Entscheidender ist diese Funktion, bei einem vorliegende Karpaltunnelsyndrom 35 und so erwähnten Sanjuan-Cervero et al. 36 , dass die URAM-Skala theoretisch beim Karpaltunnelsyndrom eingesetzt werden kann. Hensler et al. 17 wiesen darauf hin, dass weniger das Ergreifen von kleinen Gegenständen, das vorranging mit dem Daumen und Zeigefinger durchgeführt wird, problematisch ist, sondern eher das Ergreifen von größeren Gegenständen, welches ein Öffnen und Schließen der ganzen Hand erfordert, eingeschränkt ist. Die durchgeführte Faktorenanalyse von Lanfranchi et al. 14 zeigte, dass mittels URAM-Skala Funktionen evaluiert werden, die unterschiedliche Handpositionen erfordern. Item #1, #2, #5 – #8, #10 evaluieren Funktion, die mit „offener Hand“ durchgeführt werden. Lediglich 3 der 10 Items, Item #3, #4 und #9, evaluieren Aktivitäten, die eine „geschlossenen Hand“ oder eine Greiffunktion bedingen und damit verbundenen Einschränkungen aufgrund der DK aufzeigen 14 . Im Gegensatz dazu offenbarte die Faktorenanalyse von Hensler et al. 17 eine Eindimensionalität der URAM-Skala. Die Konstruktvalidität wurden meist anhand von Korrelationen zwischen der URAM-Skala und der Tubiana-Klassifizierung 17 20 30 31 , dem DASH 13 oder der SDSS 31 evaluiert. Dahingehen ist zu beachten, dass Autoren anderer Studien darauf hinwiesen, dass von den Betroffenen erst ab einem Extensionsdefizit von 91°(Tubiana 3) 37 eine Einschränkung wahrgenommen wird und daher bei geringeren Ausprägungen fast kein 38 oder kein signifikanter Zusammenhang 39 zwischen dem bestehenden Extensionsdefizit und der wahrgenommenen Einschränkungen besteht. Zusätzlich wird erwähnt, dass der DASH bei der DK nicht ausreichend valide ist 7 40 41 . Die Korrelation mit der SDSS war am höchsten 31 . In Rahmen der italienischen Validierung wurde im Item #1 das Waschen mit einem „Waschhandschuh“ als Diskrepanz gesehen, da dieser kaum zum Waschen verwendet wird 14 . In der englischen Version wurde dieses Item um den Aspekt des „keeing your hand flat“ erweitert, da zum Waschen ein „Waschhandschuh“ in Frankreich, nicht aber im Vereinigten Königreich verwendet wird 13 . In der deutschen Version wird nach der „Fähigkeit des Flachhaltens der Hand“ gefragt, aber auch der „Waschlappen“ erwähnt. Die Relevanz des „Waschlappens“ wurde bei der deutschen Validierung jedoch nicht thematisiert 16 . Der mögliche relevante Einfluss der Händigkeit oder der Verwendung einer bestimmten Hand bei Gesten (Item #4: jemanden per Handschlag begrüßen ) oder Tätigkeiten (Item #3: halten einer Flasche) wurde in den inkludierten Studien nicht untersucht. Eine Betroffenheit der dominanten Hand kann jedoch zu stärkeren Einschränkungen im Alltag führten und ein relevanter Faktor für die Inanspruchnahme einer Behandlung sein 42 . Darüber hinaus eignen sich die Betroffenen im Laufe der Zeit Kompensationsstrategien beim Greifen oder Hantieren mit den 3 radialen Finger an 32 , die einen Einfluss auf die Items #2, #3 und haben können.

Die exzellente Retest-Reliabilität bei kurzzeitigen 13 14 als auch bei langfristigen Erhebungen 20 , ist dahingehend relevant, da drei Monate nach Interventionen und darüber hinaus, Verbesserungen zu erwarten sind 43 . Als Erklärung für die mäßige mittelfristige Retest-Reliabiltät, die nach einem Monat erhoben wurde, wird der nicht äquivalente Zeitpunkt der Erhebung der Werte und dessen Beeinflussung genannt 31 . Die interne Konsistenz war exzellent 13 17 20 31 , wobei eine Redundanz nicht festgestellt wurde 13 17 .

Die Werte der SRM als auch die ES belegen eine mittlere bis hohe Responsivität 13 17 20 28 . Broekstra et al. 19 weisen darauf hin, dass mittels der URAM-Skala das Fortschreiten der DK lediglich auf Gruppenniveau und nicht bei individuellen Verläufen eruiert werden könne. Die Diskrepanz zwischen den MIC-Werten (1,5 19 –10,5 28 ) kann auf den unterschiedlichen Behandlungsoptionen und den damit verbundenen Wiederherstellungszeiten basieren 17 . Es wird auch darauf hingewiesen, dass unterschiedliche MICs den Status zu Beginn der Behandlung widerspiegeln können 17 . Aufgrund der hohen Spezifizität von 86–88% 19 28 kann die URAM-Skala die Einschränkungen resultierend aus der charakteristischen Kontraktur und der damit einhergehenden Veränderungen der Handfunktion im Krankheitsverlauf messen 13 19 30 . Im Gegensatz dazu weisen die Ergebnisse der Metaanalyse von Sanjuan-Cervero et al. auf eine hohe Sensitivität (80,23%) und niedrige Spezifizität (2,6%) der URAM-Skala hin. Die niedrige Spezifizität basiert dabei auf unterschiedlichen Faktoren, wie das das Fehlen von Definitionen von Schwellenwerten oder der Uneinigkeit der Grenze von Krankheit oder Rezidiv 36 . Als Erklärung für die hohe Sensitivität wird die Tatsache gesehen, dass einige Fragen der URAM-Skala die Handmobilität thematisieren 36 , und Studien 13 14 zeigen, dass der URAM-Wert eine starke Korrelation mit der Tubiana-Klassifizierung aufweist. Hensler et al. 17 weisen zudem auch darauf hin, dass bei der Interpretation unterschiedliche Behandlungen und daraus resultierende Erholungszeiten 44 , die diesen Werten zugrunde liegen, zu beachten sind. Zum anderen wurden unterschiedlichen Techniken, Hierarchical Summary Receiver Operating Characteristic (HSROC) 36 sowie die receiver operating characteristic (ROC) Kurve 19 28 , zur Kalkulation der Interpretation herangezogen.

Aus folgenden Gründen kann in der Indikationsstellung oder der Behandlung der DK die URAM-Skala von Vorteil sein. Mit der Konstruktvalidität verhilft die URAM-Skala den involvierten Professionen den Einfluss der DK auf den Alltag und funktionellen Auswirkungen des Patienten zu verstehen, und die damit einhergehenden Beschwerden im Verlauf der Erkrankung oder am Beginn der Behandlung zu erfassen 7 14 17 . Aufgrund der mittlere bis hohe Responsivität wird die Verwendung der URAM-Skala als passendes und vorzuziehendes Instrument zur Messung von Behandlungseffekten im klinischen Alltag, in Registern oder in Studien empfohlen 12 17 . Da zum Ausfüllen der URAM-Skala aufgrund ihrer Länge weniger als zwei Minuten benötigt werden, ist sie im Klinikalltag gut anwendbar 14 17 .

Diese Arbeit weist einige Limitationen auf. So wurden lediglich Publikationen in englischer oder deutscher Sprache berücksichtigt. Daher besteht die Möglichkeit, dass nicht alle existierenden Publikationen, die die URAM-Skala und deren Gütekriterien thematisieren, eingeschlossen werden konnten. Eine Überprüfung der Datenauswertung durch eine weitere Person, um eine Minimierung von potenziellen Bewertungsbias zu erreichen, konnte nicht durchgeführt werden, da es sich hierbei um einen Teilaspekt des PhD-Projektes der Autorin handelt. Weiters wurde diese Arbeit von einer Person, der Autorin, verfasst und deren Interpretation kann daher von anderen abweichen.

## Schlussfolgerung

Die Ergebnisse dieser Übersichtsarbeit zeigen, dass die Gütekriterien der URAM-Skala kaum erforscht sind. Dennoch verfügt der erste DK-spezifische Fragebogen über eine gute Konstruktvalidität, eine exzellente Retest-Reliabilität, eine exzellente Interne Konsistenz und über eine mittlere bis hohe Responsivität. Aufgrund ihrer Kürze ist die Akzeptanz der URAM-Skala gegeben und gut im Klinikalltag einsetzbar. In Kombination mit anderen objektiven Erhebungsinstrumenten verhilft dieser erste diagnose-spezifische Fragebogen dazu, den Einfluss der Erkrankung auf die Handfunktion zu erfassen, die Betroffenen zu verstehen und daraus resultierende Entscheidungen bezüglich Behandlungszeitpunkt zu treffen, sowie Behandlungseffekte bei der DK zu beurteilen.
